# A New View of Diabetes

**Published:** 1908-06-13

**Authors:** 


### The Hospital

A Journal of

The Medical Sciences and Hospital Administration.

New Series. No. 65 Vol. III. [No. 1137, Vol. XLIV.] SATURDAY, JUNE 13, 1908.

### A New View of Diabetes.

Dr. R. G. Eccles, of Brooklyn,* is a confirmed teleologist. We can perhaps best express his attitude of mind by transcribing some quotations of his from a speech made by Sir Frederick Treves in 1905, for he subscribes to them unreservedly. The quotations are as follows: "The processes of disease are aimed, not at the destruction of life, but at the saving of it." "If it were not for 'disease,' in the popular sense, the human race would soon be extinct." "For centuries there has been presented the strange spectacle of men of science struggling to the utmost to thwart, to curb, to annihilate a process of cure." Proceeding to amplify these propositions, with which he wholly identifies himself, Dr. Eccles instances the tendency of the older medicine towards opposing natural indications for treatment. For example, we are told of the practice of withholding water from choleraic patients, and from patients parched with fevers, and are reminded that inflammation, whose suppression at the earliest possible moment was the goal of treatment, is now known to be a curative process and one to be promoted. With this preamble we are faced with the proposition that in diabetes the "excessive outpour of sugar may be an attempt on the part of nature to remove some obstruction, cure some injury, or rid the body of some irritant." Confronted with our regrettable ignorance touching glycosuria in general, we are invited to attempt new paths. "Past experience with fever and inflammation ought to have rid our minds of the obsessing notion that any general symptom of a disease is an evil to be curbed. Why not try the Darwinian idea for a while, and assume that the sugar is being wasted as a physiological reaction to some present irritant of exogenous origin?" In support of this thesis the author calls the fact that the formation of dextrose will continue in diabetics even when all carbohydrate food is withdrawn sure evidence that the body is crying for the presence of dextrose in the blood, and that the sudden withdrawal of carbohydrates is in itself an exciting cause of diabetic coma, while the onset of coma synchronises with the disappearance of sugar from the urine. This means, according to the author, that "in the collapse of the body the effort to hold a large amount of sugar in the circulation is withdrawn, and glycolysis proceeds unhindered. The physiological effort at saving the body from the impending danger of the real cause of diabetes is compelled to end by the more imminent danger of the coma, perhaps brought about by false feeding." What evidence is there, we are asked, that the presence of sugar in the blood in excess is in itself harmful? Have not credible authorities reported instances of persistent glycosuria, without any sign of impairment of health, extending over periods of seventeen years or more? While enormous quantities of dextrose have been administered subcutaneously to dogs without producing any signs of poisoning. Sugar-eating populations, we are told, do not suffer from diabetes, which is a disease of the proteid-feeders of higher civilisations. The conclusion of the matter is that the excessive production of sugar in glycosurics is a protective process, and its administration in quantity their only safeguard against serious mischance.

It is always wholesome to find old problems attacked from new standpoints, for there is no question that humanity in general is much too prone to be ridden by tradition and authority. Of the existence of this liability the examples of inflammation and the treatment of the thirst of fever, cited by the author, are effective illustrations. From time to time there arise among us new prophets expounding large opinions which shall revolutionise the conceptions of the time, and since it is impossible to foretell the arrival of a Darwin or a Pasteur, we cannot afford to turn them a deaf ear. But we confess frankly that Dr. Eccles has not convinced us. In the first place he appears to be labouring under a confusion of thought when he allies himself with Sir Frederick Treves in the view that "if it were not for 'disease,' in the popular sense, the human race would soon be extinct." The words are too vague to be given a meaning. What is disease in the popular sense? Is it the diathesis which causes a man to develop a stone in his kidney? Or the pain he feels when a portion of the stone becomes detached and, entering his ureter, subjects him to the agonies of renal colic? Or is it the entrance of the bacillus coli communis into the fluid of the resulting hydronephrosis, by which means this

* "Med. Record," N.Y., May 9, 1908.

liquid is converted into pus? And are any of these to be considered as life-saving devices? Clearly we need some more definite basis for our conceptions, since this will not do. Yet the most notable feature of Dr. Eccles' article is his lack of definition. He does not even tell us what he means by diabetes, and his references to glycosuria would seem to imply that his view is comprehensive enough to include every variety of sugary urine. But if we confine ourselves for the moment to instances of definite diabetes, a little reflection displays the kind of *impasse* into which our author's reasoning will lead. An individual develops within himself " some obstruction "—delightful phrase!—which demands for its removal the presence in excess of sugar in the blood. This sugar must come from somewhere, so kindly nature decrees that the patient shall evolve a prodigious appetite to meet the call. But nature is in an extravagant mood; a large part of the sugar for which she has thus made provision is wasted by way of the kidneys, and incidentally involves the subject of her experiments in an invincible thirst. It is no matter. Hunger, thirst, and polyuria, these are all for the best, and the physician's duty is to maintain them all at their highest pitch, for there is no other safeguard from catastrophe. No! Decidedly we cannot compete in optimism with Dr. Eccles. Without pretending to explain on other grounds the apparent induction of coma by sudden withdrawal of all carbohydrates, or the cessation of glycosuria with the onset of coma, or the formation of sugar by the katabolism of proteids, we cannot profess acceptance of the solution he offers us. Indeed it seems better to admit them still as mysteries than to expose our mental digestions to the risks entailed by this new hypothesis.

